# Supplementary material for: Quantifying fair income distribution in Thailand
Source: PLoS One. 2024 Apr 4;19(4):e0301693. doi: 10.1371/journal.pone.0301693 (PMC10994331; doi:10.1371/journal.pone.0301693)
Supplement: S1 Table — (PDF) [file pone.0301693.s001.pdf]

Supporting Information for

Quantifying fair income distribution in Thailand

Thitithep Sitthiyot[1*], Kanyarat Holasut[2]

[1] Department of Banking and Finance, Faculty of Commerce and Accountancy, Chulalongkorn University, Bangkok, Thailand

[2] Department of Chemical Engineering, Faculty of Engineering, Khon Kaen University, Khon Kaen, Thailand

[*] Corresponding author

E-mail: thitithep@cbs.chula.ac.th (TS)

S1 Table. The calculated values of L, H, μ and α as well as the estimated values of W and β^{*} based on PK method.

Year	L	H	μ	α	W	β^{*}
1988	10.216	37.496	23.856	0.220	32.108	0.014
1990	9.621	38.234	23.927	0.210	32.394	0.013
1992	9.081	38.940	24.011	0.201	32.635	0.012
1994	9.512	38.454	23.983	0.207	32.370	0.013
1996	9.691	38.218	23.955	0.210	32.293	0.013
1998	9.875	37.976	23.925	0.214	32.195	0.013
2000	9.394	38.631	24.013	0.205	32.413	0.013
2002	9.873	38.034	23.954	0.213	32.149	0.013
2004	10.240	37.522	23.881	0.220	32.007	0.014
2006	9.901	38.202	24.052	0.212	31.906	0.014
2007	10.246	37.651	23.948	0.219	31.847	0.014
2009	10.454	37.335	23.894	0.223	31.775	0.014
2011	10.718	36.979	23.849	0.228	31.582	0.014
2013	11.254	36.664	23.959	0.236	30.930	0.015
2015	11.675	35.866	23.770	0.248	30.968	0.015
2017	11.440	36.052	23.746	0.244	31.258	0.015
2019	11.963	35.284	23.623	0.257	31.113	0.016
2021	11.944	35.306	23.625	0.257	31.088	0.016
