# Supplementary material for: Quantifying fair income distribution in Thailand
Source: PLoS One. 2024 Apr 4;19(4):e0301693. doi: 10.1371/journal.pone.0301693 (PMC10994331; doi:10.1371/journal.pone.0301693)
Supplement: Attachment — Submitted filename: Response to Reviewers_R1.pdf [file pone.0301693.s002.pdf]

Response to Reviewers

Manuscript Number: PONE-D-23-25866R1

Title: Quantifying fair income distribution in Thailand

We sincerely thank Reviewer #1 and Reviewer #2 for providing several important comments, concerns, and suggestions. We would like to inform both Reviewers that we have made a substantial revision according to comments, concerns, and suggestions made by both Reviewers. We hope that our revised manuscript is clearer in all aspects that Reviewer #1 and Reviewer #2 have commented, concerned, and suggested. Let us respond to both Reviewers' comments, concerns, and suggestions as follows.

Reviewer #1

This study examines income inequality in Thailand over the past three decades and determines whether it is fair. It also defines what fair inequality should be in Thailand. The study uses fairness benchmarks based on distributive and procedural justice to quantify fair inequality. The results show that the bottom 20%, second 20%, and top 20% of income earners receive more than their fair share, while the third 20% and fourth 20% receive less than their fair share. These findings can be used to evaluate the effectiveness of income redistribution measures and to formulate policies aimed at achieving fair income distribution in Thailand. The study also demonstrates that fairness benchmarks can be used as a practical guideline for designing income redistribution policies and measures. Below I have some suggestions.

- Regarding Fig. 2, it would be beneficial for the authors to provide a more explicit rationale for their interpretation of the scatter plots. The current presentation lacks clarity on why the data align with the distribution or fit lines, and additional details would enhance the persuasiveness of this analysis.

In response to Reviewer #1's comment with regard to the interpretation of results as shown in Fig. 2, we would like to inform Reviewer #1 that we explain the rationale for the interpretation of scatter plots of actual quintile income shares relative to their corresponding fairness benchmarks in Materials and Methods, paragraphs 5 and 6 which we use it as a basis for interpreting our results as provided in Results, paragraphs 2, 3, and 4 in our revised manuscript.

- I recommend conducting a separation analysis, specifically comparing the top 20% and bottom 20% data distributions. This would offer valuable insights into potential variations in fairness and income across different groups. Highlighting the distinctions between these two extremes could contribute significantly to the study's comprehensiveness.

We would like to thank Reviewer #1 for this useful suggestion. We add the discussion on variations in unfairness in income distribution across different population groups in Results, paragraphs 5 and 8 in our revised manuscript.

- It is essential to articulate the significance of this study. Why is it important, and what broader implications does it hold? Additionally, consider discussing the fairness of income distribution in the context of other countries and exploring any relationships that may emerge. Providing this contextualization will enrich the paper and strengthen its contribution to the field.

In response to Reviewer #1's suggestion on articulating the significance of our study and its implications, we state the significance of our study in Introduction, paragraphs 3 and 9, and in Discussion, paragraph 1 in our revised manuscript.

The implications of our study are also provided in Results, paragraphs 9 – 11 and in Discussion, paragraphs 6 – 8 in our revised manuscript.

In addition, we discuss the fairness in income distribution in the context of other countries and explore the similarity and/or difference among them in Discussion, paragraphs 2 and 3 in our revised manuscript as suggested by Reviewer #1.

Reviewer #2

This paper contributes to the literature on fair income distribution by empirically applying the method developed by Sitthiyot and Holasut (2022) in Thailand. While the analysis of alternative measures to evaluate inequality levels is crucial for understanding the magnitude of unfair inequalities, the paper has several drawbacks that need attention before I can recommend it for publication. In the following sections, I will outline the main limitations of the study in the hope that these points will assist the authors in enhancing their analysis.

(1) There exists a vast literature on unfair inequality, and I recommend the inclusion of additional references to provide a more comprehensive theoretical and normative framework for measuring the unfair distribution of income. Notable works in this field include Almås et al (2011), Cappelen, A. W., & Tungodden (2017), and Hufe et al. (2022). While the authors acknowledge contributions from the econophysics stream, it is essential to recognize the more traditional economic literature, which has also significantly contributed to the understanding of income distribution.

We sincerely thank Reviewer #2 for this suggestion. We would like to inform Reviewer #2 that we include studies by Almås et al. (2011), Cappelen and Tungodden (2017), and Hufe et al. (2022) in Introduction, paragraph 3 in our revised manuscript. We do learn a lot from these studies.

(2) The authors chose to exclusively apply the method they developed in a previous paper (Sitthiyot and Holasut, 2022). While this choice is valid, it is crucial to complement this analysis with other approaches used by previous studies in order to validate the robustness of the conclusions. A comparative analysis of different methods would strengthen the paper's contribution to the field.

We would like to thank Reviewer #2 for this suggestion, in our revised manuscript, we conduct a comparative analysis by using the method for estimating optimal income distribution representing feasible fair equality developed by Park and Kim (2021) in order to provide an alternative recommendation for designing policies aimed to achieve fair income distribution. The main reason for choosing Park and Kim (2021)'s method is due to the availability of data of Thailand that can be used to conduct the analysis. We would like to

inform Reviewer #2 that we report the calculated and estimated values of all parameters based on Park and Kim (2021)'s method in S1 Table in Supporting Information.

(3) The paper lacks an explanation of how Equations (1) – (5) are derived. Although it is assumed they originate from Sitthiyot and Holasut (2022), no reference to this is made in the text. The authors should clarify how these equations are obtained, the data used for estimation, and how this data compares with the dataset used in the empirical application developed in this paper.

In response to Reviewer #2 on this issue, we would like to inform Reviewer #2 that we provide the derivation of Equations (1) – (5) and the data used for estimation in Materials and Methods, paragraphs 1 – 4 in our revised manuscript. We also cite our work (Sitthiyot and Holasut, 2022) in the text and provide reference in our revised manuscript. In addition, the dataset used for empirical analysis is stated in Materials and Methods, paragraph 12 and in References in our revised manuscript.

(4) Considering the period covered (1988 – 2021), it is questionable whether Eqs. (1) to (5) can accurately measure the fair distribution over this extended timeframe. Changes in the income distribution of athletes, considered the fair distribution in this context, may have occurred over the last 30 years. The analysis should account for these potential changes to enhance the temporal validity of the study.

We totally agree with Reviewer #2 on this issue and would like to thank Reviewer #2 for pointing this out. For Eqs. (1) – (5) to be used for measuring fair income distribution, we make an assumption that the distributions of professional athletes' salaries are stable across time. We state this in Materials and Methods, paragraph 4 and in Discussion, paragraph 2 in our revised manuscript.

(5) The main conclusion asserting that the bottom 40 per cent owns more income than what would be deemed fair is a strong statement. Given the potential policy implications, such as redistributing income from these quantiles to the top, the authors should address the concern that this could exacerbate poverty levels in a country where 6.3 per cent of the population is already classified as poor. A more nuanced discussion of the potential consequences is warranted.

We would like to sincerely thank Reviewer #2 for addressing this important point. In response to concern and suggestion made by Reviewer #2 on this issue, we would like to inform Reviewer #2 that we have made a major adjustment in our revised manuscript by seriously taking Reviewer #2's concern and suggestion into account and rewriting policy implication based on our results regarding the bottom 40% by using the actual quintile income shares and the income Gini index of Thailand in 2015 as a case study.

Given the goal is to achieve the income Gini index of 0.410 as specified in Thailand's Twelfth National Economic and Social Development Plan, policymakers could use the information on fair quintile income shares based on the fairness benchmarks for designing policy in order to not only lower income inequality but also achieve fair income distribution across all population groups at the same time by redistributing income from the top 20% to the third 20% and the fourth 20% while the income shares of the bottom 20% and the second 20% are mostly unaffected. We discuss all of these in Discussion, paragraphs 6 and 7 in our revised manuscript and also use Fig. 6 to supplement our discussion on this issue.
