# Supplementary material for: Quantifying fair income distribution in Thailand
Source: PLoS One. 2024 Apr 4;19(4):e0301693. doi: 10.1371/journal.pone.0301693 (PMC10994331; doi:10.1371/journal.pone.0301693)
Supplement: Attachment — Submitted filename: Response to Reviewers_R2.pdf [file pone.0301693.s003.pdf]

Response to Reviewers

Manuscript Number: PONE-D-23-25866R2

Title: Quantifying fair income distribution in Thailand

We sincerely thank all three Reviewers for providing a number of important comments and making clear suggestions with the view of improving the quality and clarity of our paper. We have addressed all of the Reviewers' and the Academic Editor's comments and tried our best to incorporate all comments and suggestions into our revised manuscript. We hope that our revised paper is clearer in all aspects that the Reviewers and the Academic Editor have commented and suggested. Let us respond to each Reviewer's and the Academic Editor's comments and suggestions as follows.

Reviewer #3

1. It is necessary to refine the abstract. In the current state the paper, due to the language it is very challenging to understand the narrative. It is need to specify: the initial data, the hypothesis of the study to strengthen, specify the scope of application, limitations/directions of future research. For example, How to understand this "Given growing concern about high income inequality in Thailand as opposed to empirical findings around the world showing people's preference for fair income inequality over unfair income equality, it is therefore important to examine whether inequality in income distribution in Thailand over the past three decades is fair, and what fair inequality in income distribution in Thailand should be"?

We sincerely thank Reviewer #3 for this comment. In our revised manuscript, we rewrite Abstract as suggested by Reviewer #3.

2. I believe there are findings from the context of your study that will have interest for scholars and policy makers. However, the purpose of the study is formulated very incorrectly and it should be refined.

We would like to sincerely thank Reviewer #3 for finding our paper useful. In our revised manuscript, we rewrite the purpose of the study in Abstract, Introduction, paragraph 3, and Discussion, paragraph 1.

3. The introduction should outline the context of the problem and justify the need for research (research gap, a new phenomenon).

In response to Reviewer #3 on this issue, we have made a substantial revision by rewriting the whole Introduction section in our revised manuscript.

4. Unfortunately, I did not understand the literature review. Literature review is also completely chaotic. Why is it dedicated? It should be fully subordinated to the coverage of

the results of previous scientific research on the subject, which will be determined in the purpose of your research.

We would like to sincerely thank Reviewer #3 for this comment. In Introduction, paragraphs 6 and 8-12, in our revised manuscript, we have made a major revision by rewriting the literature review as suggested by Reviewer #3 and the Academic Editor.

5. The methods should be described as concretely as possible. The section may include a description of data, methods of collecting, etc.

In response to Reviewer #3 on this issue, we would like to inform Reviewer #3 that the previous version of our method was shorter than the current one. The current version is the product based on comments made by Reviewer #2 who, in the first round of review, asked us to explain in details how equations for fairness benchmarks are derived and also to conduct a comparative analysis by using different methods of measuring fair income distribution. In addition, we would like to inform Reviewer #3 that the description of data used in our study and how we obtained them are described in Methods, paragraph 12 and in References in our revised manuscript.

Reviewer #4

- I was not one of the first-round reviewers so this was the first time I read the paper. I acknowledge the professional and rigorous approach you have taken to answering the previous reviewers. I really appreciate the opportunity to read this paper and I can see there are some valuable ideas within it. Unfortunately, I am unable to recommend acceptance and I do not think even a thoroughly revised version of this paper would be suitable for PLOS-ONE.

I enjoyed trying to get to grips with the intuition underlying this paper. What you have argued in previous work is that for whatever GINI is deemed acceptable, there is a curve that selects the approximate distribution between income quintiles that would emerge from competition based on fair and transparent rules, using professional sports rules and their resulting income distributions as the baseline for this judgement. I think there is a lot that could be said to challenge this baseline but it is clever and aligns with the way people are generally unconcerned with inequality that emerges between sports people competing under the same rules.

In this paper, you apply this to evaluating the income distribution of Thailand and show how the distribution between quintiles would have to change whether following your baseline judgement or Park and Kim's (2021) more demanding approach to tackling inequality.

The key question is how this develops your previous work as, on its face, 'A quantitative method for benchmarking fair income distribution' makes exactly the same point but, more ambitiously, for 75 countries. I cannot detect a compelling answer. The new paper's contribution is to consider Thailand's GINI over 30 years rather than a single year snapshot. That is potentially very interesting but the paper discusses very little of the

changing economic structure or policy over that thirty year period. In terms of analysis, it might be as well be 30 (pretty similar) statistical snapshots, each independently judged against two normative baselines, rather than a narrative that might indicate what sort of policies might make an income distribution fairer. What sort of rules and economic institutions are Thai people subject to and how do they differ from the fair and transparent rules of professional sports? We learn very little about this.

In response to Reviewer #4 on this issue, we would like to clarify with Reviewer #4 that the main objective of our previous paper is to introduce a new quantitative method for measuring fair income distribution. The reason that we use the data of 75 countries from the World Bank is mainly to demonstrate how our method works for various countries instead of focusing on one specific country.

For the current manuscript, we show how the method that we previously developed can be applied to analyze fair income distribution across time using Thailand as a case study. We do acknowledge the importance of rules and economic institutions the Thai people are subject to and how they differ from the fair and transparent rules of professional sports but this topic is beyond the scope of our study and worth for future investigation.

- Critically, one could imagine policies that arrange income into the appropriate quintiles but without being fair and transparent. So, the outcomes might look 'fair' but they would not be fair as people experience them. I reminded of points made by Acemoglu and Robinson reviewing Piketty where they point out the changing distributions of South Africa and Sweden look similar but one is based on exploitative institutions whereas the others are remarkably fair. There is not more going on to judge a process than a particular shape of the outcomes:

Acemoglu, D., & Robinson, J. (2015). The Rise and Decline of General Laws of Capitalism. Journal of Economic Perspectives, 29(1), 3–28.

On this account, if the proposed distribution were made a simple 'target' to hit, policymakers could still easily fail to create outcomes that people within the 'game' policymakers have set up would judge as fair. So overall the addition of the temporal element does not seem to have expanded the analysis in a compelling or insightful on top of what you have already done.

In response to Reviewer #4's comment on this issue, we state in Discussion, paragraph 9 in our revised manuscript that, in order to achieve fair income distribution for a particular value of income Gini index, policymakers have to design and implement income distribution policies on the bases of procedural justice and distributive justice similar to those in professional sports such that the Thai population across socio-economic groups would unconditionally and whole-heartedly perceive as fair.

- I have a more prosaic concern with regards to the data. In your previous paper (Appendix Table A1), Thailand is listed as having a GINI index of 0.360. This is pretty close to the World Bank Databank's most recent estimate of 0.351 so it seems to be correct in that paper. That also chimes with the Asian Development Bank's narrative:

'Thailand's GINI coefficient on the expenditure side fell from a peak of 0.48 in 1992 to 0.35 in 2019.'

https://www.adb.org/sites/default/files/linked-documents/tha-cps-2021-2025-ld01.pdf

Yet, in the new paper the GINI is always 0.43 or above. The paper specifies that this is the income GINI (does not discuss expenditure explicitly) but does this suggest that the expenditure GINI used in the previous paper (and elsewhere) was a misapplication? Why the shift to a different series for this paper? This matters since the premise for the focus on Thailand is that it is remarkably unequal compared to other countries in the region. But according to the WorldBank, it is not, or at least not so much anymore. We would need to see income GINIs for the other countries to know this was a case worth exploring the in the first instance.

We would like to sincerely thank Reviewer #4 for your comment on the use of data on Gini index from the World Bank in our previous paper. We would like to inform Reviewer #4 that we used the data on Gini index from the World Bank on the premise that the data come from the same source and they are the "World Bank estimate". Below are examples of the information with regard to the data on Gini index of Thailand and other countries in Excel format that we downloaded from the World Bank website back in early 2020.

Country Name	Country Code	Indicator Name	Indicator Code
Thailand	THA	GINI index (World Bank estimate)	SI.POV.GINI
Czech Republic	CZE	GINI index (World Bank estimate)	SI.POV.GINI
Germany	DEU	GINI index (World Bank estimate)	SI.POV.GINI
Djibouti	DJI	GINI index (World Bank estimate)	SI.POV.GINI
Dominica	DMA	GINI index (World Bank estimate)	SI.POV.GINI
Denmark	DNK	GINI index (World Bank estimate)	SI.POV.GINI
Dominican Republic	DOM	GINI index (World Bank estimate)	SI.POV.GINI

Source: https://data.worldbank.org/indicator/SI.POV.GINI [Accessed 7 March 2020].

In response to Reviewer #4's comment on the issue whether Thailand still has high income inequality, we would like to inform Reviewer #4 that, according to the World Bank (2022) which we cite this report in Introduction, paragraph 1 in our revised manuscript, Thailand has the highest income inequality in East Asia in 2019.

The World Bank. 2022. Thailand Rural Income Diagnostic: Challenges and Opportunities for Rural Farmers. https://www.worldbank.org/en/country/thailand/publication/thailand-rural-income-diagnostic-challenges-and-opportunities-for-rural-farmers.

In addition to the issue whether income inequality in Thailand is still high as noted by Reviewer #4, we would like to inform Reviewer #4 that the more important issue which is the main objective of our study is to investigate whether income inequality in Thailand is fair by using the fairness benchmarks introduced in our previous paper. Even though Thailand were

considered a low income inequality country, it would still be worthwhile to explore whether income distribution in Thailand is fair. This is because it is possible that a country has low income inequality but the distribution of income in that country is not fair.

In response to Reviewer #4's question regarding the use of data on income shares by quintile and income Gini index from the Office of National Economic and Social Development Council of Thailand (NESDC), we would like to inform Reviewer #4 that since we investigate fair income distribution in Thailand, it would be appropriate to use the data from the NESDC which is the government's official source that provides the data on Thailand's income statistics.

Although the NESDC website has an English language version, the income data can be accessed from the NESDC's website in Thai language version only. The link to the NESDC website is https://www.nesdc.go.th/main.php?filename=PageSocial. If readers go to this link and click "สถิติด้านความยากจนและการกระจายรายได้" (in Thai) which can be translated as "Poverty and income distribution statistics", readers can download the data on income shares by quintile and income Gini index of Thailand from 1988 to 2021 which are in Tables 8.1 and 8.2. The step-by-step of how to access the data on quintile income share and income Gini Index is shown below.

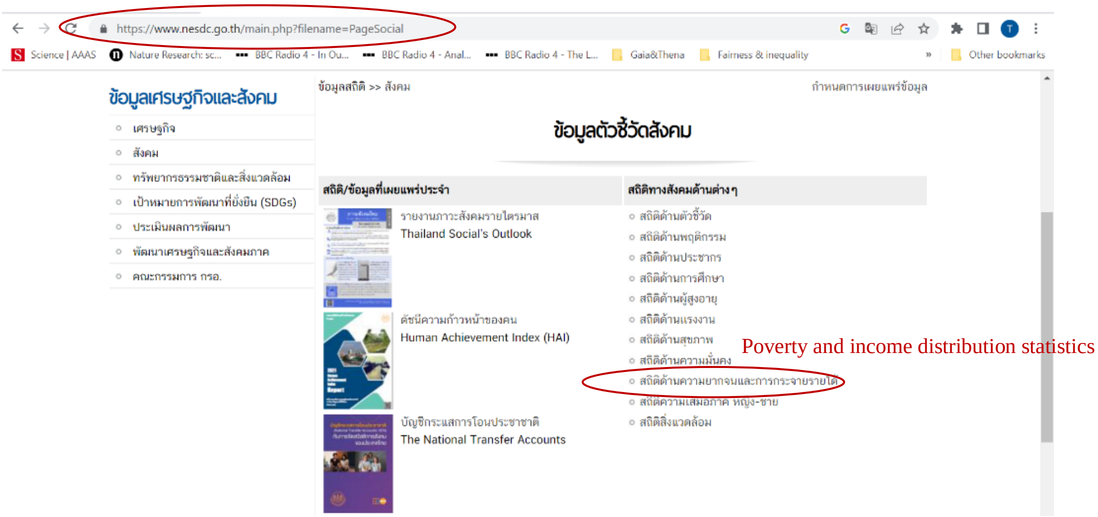

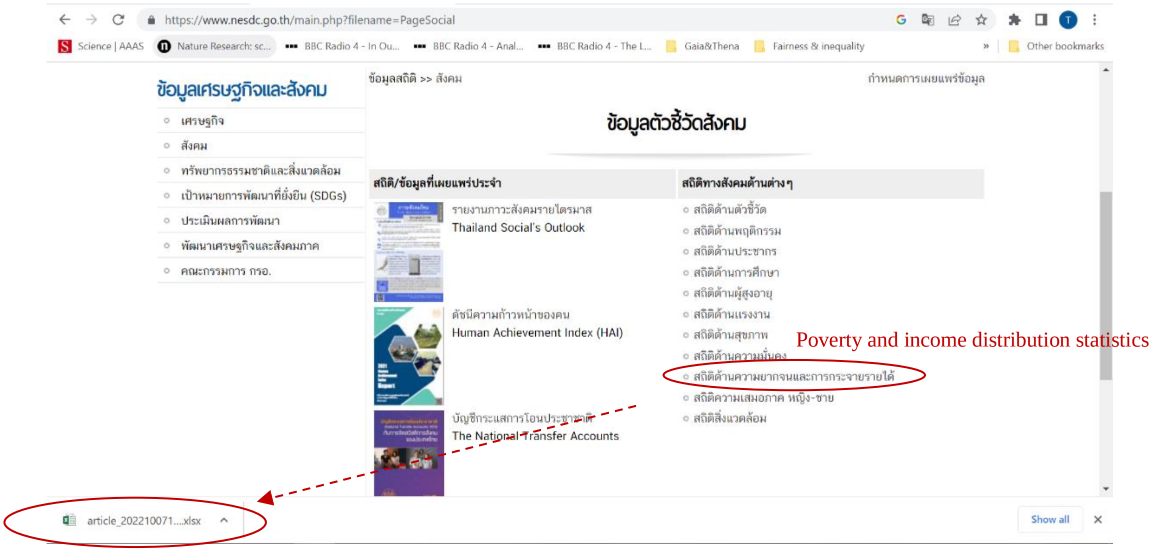

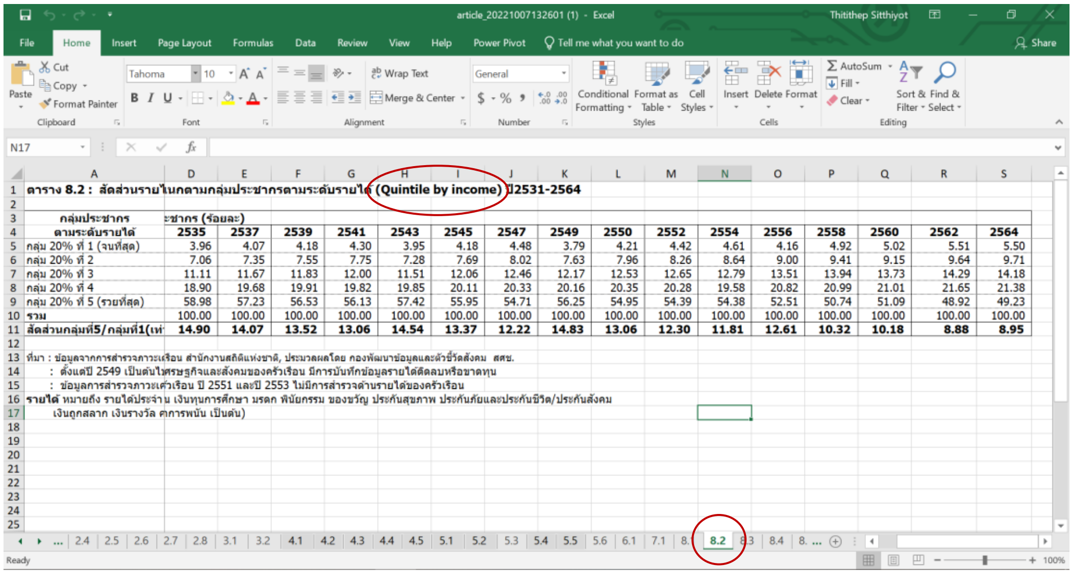

ตาราง 8.2 : สัดส่วนรายได้ในกลุ่มประชากรตามระดับรายได้ (Quintile by income) ปี2531-2564

กลุ่มประชากร ตามระดับรายได้	2535	2537	2539	2541	2543	2545	2547	2549	2550	2552	2554	2556	2558	2560	2562	2564
กลุ่ม 20% ที่ 1 (จนที่สุด)	3.96	4.07	4.18	4.30	3.95	4.18	4.48	3.79	4.21	4.42	4.61	4.16	4.92	5.02	5.51	5.50
กลุ่ม 20% ที่ 2	7.06	7.35	7.55	7.75	7.28	7.69	8.02	7.63	7.96	8.26	8.64	9.00	9.41	9.15	9.64	9.71
กลุ่ม 20% ที่ 3	11.11	11.67	11.83	12.00	11.51	12.06	12.46	12.17	12.53	12.65	12.79	13.51	13.94	13.73	14.29	14.18
กลุ่ม 20% ที่ 4	18.90	19.68	19.91	19.82	19.85	20.11	20.33	20.16	20.35	20.28	19.58	20.82	20.99	21.01	21.65	21.38
กลุ่ม 20% ที่ 5 (รวยที่สุด)	58.98	57.23	56.53	56.13	57.42	55.95	54.71	56.25	54.95	54.39	54.38	52.51	50.74	51.09	48.92	49.23
รวม	100.00	100.00	100.00	100.00	100.00	100.00	100.00	100.00	100.00	100.00	100.00	100.00	100.00	100.00	100.00	100.00
**สัดส่วนกลุ่มที่5/กลุ่มที่1(เท่**	**14.90**	**14.07**	**13.52**	**13.06**	**14.54**	**13.37**	**12.22**	**14.83**	**13.06**	**12.30**	**11.81**	**12.61**	**10.32**	**10.18**	**8.88**	**8.95**

ที่มา : ข้อมูลจากการสำรวจภาวะเศรษฐกิจและสังคมของครัวเรือน สำนักงานสถิติแห่งชาติ, ประมวลผลโดย กองพัฒนาข้อมูลและตัวชี้วัดสังคม สศช.
: ตั้งแต่ปี 2549 เป็นต้นไปเศรษฐกิจและสังคมของครัวเรือน มีการบันทึกข้อมูลรายได้ติดลบหรือขาดทุน
: ข้อมูลการสำรวจภาวะเศรษฐกิจครัวเรือน ปี 2551 และปี 2553 ไม่มีการสำรวจด้านรายได้ของครัวเรือน
รายได้ หมายถึง รายได้ประจำ เงินทุนการศึกษา มรดก พินัยกรรม ของขวัญ ประกันสุขภาพ ประกันภัยและประกันชีวิต/ประกันสังคม
เงินถูกสลาก เงินรางวัล การพนัน เป็นต้น)

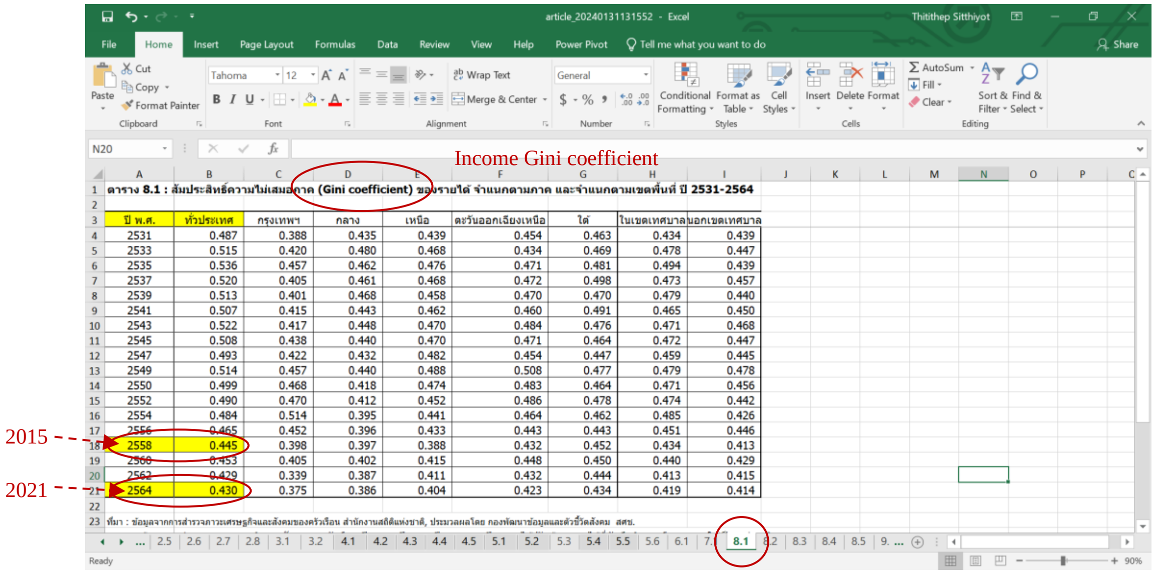

ตาราง 8.1 : สัมประสิทธิ์ความไม่เสมอภาค (Gini coefficient) ของรายได้ จำแนกตามภาค และจำแนกตามเขตพื้นที่ ปี 2531-2564

ปี พ.ศ.	ทั่วประเทศ	กรุงเทพฯ	กลาง	เหนือ	ตะวันออกเฉียงเหนือ	ใต้	ในเขตเทศบาล	นอกเขตเทศบาล
2531	0.487	0.388	0.435	0.439	0.454	0.463	0.434	0.439
2533	0.515	0.420	0.480	0.468	0.434	0.469	0.478	0.447
2535	0.536	0.457	0.462	0.476	0.471	0.481	0.494	0.439
2537	0.520	0.405	0.461	0.468	0.472	0.498	0.473	0.457
2539	0.513	0.401	0.468	0.458	0.470	0.470	0.479	0.440
2541	0.507	0.415	0.443	0.462	0.460	0.491	0.465	0.450
2543	0.522	0.417	0.448	0.470	0.484	0.476	0.471	0.468
2545	0.508	0.438	0.440	0.470	0.471	0.464	0.472	0.447
2547	0.493	0.422	0.432	0.482	0.454	0.447	0.459	0.445
2549	0.514	0.457	0.440	0.488	0.508	0.477	0.479	0.478
2550	0.499	0.468	0.418	0.474	0.483	0.464	0.471	0.456
2552	0.490	0.470	0.412	0.452	0.486	0.478	0.474	0.442
2554	0.484	0.514	0.395	0.441	0.464	0.462	0.485	0.426
2556	0.465	0.452	0.396	0.433	0.443	0.443	0.451	0.446
2558	0.445	0.398	0.397	0.388	0.432	0.452	0.434	0.413
2560	0.453	0.405	0.402	0.415	0.448	0.450	0.440	0.429
2562	0.429	0.339	0.387	0.411	0.432	0.444	0.413	0.415
2564	0.430	0.375	0.386	0.404	0.423	0.434	0.419	0.414

ที่มา : ข้อมูลจากการสำรวจภาวะเศรษฐกิจและสังคมของครัวเรือน สำนักงานสถิติแห่งชาติ, ประมวลผลโดย กองพัฒนาข้อมูลและตัวชี้วัดสังคม สศช.

Reviewer #5

- This manuscript presents a new methodology for assessing the fairness of income distribution in Thailand. It utilizes the concepts of distributive and procedural justice to quantify what is considered fair inequality, analyzing whether specific income brackets are receiving more or less than their fair share. This research could aid in evaluating the effectiveness of income redistribution policies and in formulating strategies for achieving fair income distribution.

Following the suggestions of Reviewers 1 and 2, the authors have made significant improvements to the paper. These enhancements include clarifying the study's importance

and implications, as well as providing detailed explanations of the research methodology and data.

Additional Reviewer Comments:

However, the following further improvements are necessary:

Clear Definition of Distributive Justice Required: The paper mentions distributive justice but lacks a specific definition or theoretical basis. Since various definitions and interpretations of distributive justice exist, the authors must clarify which definition they are using and the rationale behind this choice. This clarification is important for understanding the research methodology and conclusions.

In response to Reviewer #5's comment on the lack of definition of distributive justice, we provide the definition of distributive justice in Introduction, paragraph 9 in our revised manuscript.

- Improvement in Graphics Resolution: The resolution of graphs needs to be enhanced to more clearly convey the data and analysis results. This improvement is vital for effectively communicating the research findings.

We would like to inform Reviewer #5 that we prepared all figures by using a tool called PACE as recommended in *PLOS ONE*'s guideline for preparing figure. According to our understanding, the resolution of graphs is not clear because it is converted into PDF format when our manuscript was sent out for peer review.

- The methodology and results proposed in this paper have the potential to enhance the understanding of fair income distribution and could provide valuable insights to academia and policy-makers. Considering the above aspects, this manuscript appears to have sufficient potential for publication in PLOS ONE.

We would like to sincerely thank Reviewer #5 for finding our manuscript useful and recommending it for publication in *PLOS ONE*.
